# Editorial Announcement

**DOI:** 10.1186/s40709-020-00113-6

**Published:** 2020-02-19

We would like to thank all those listed below for taking the time to review for the *Journal of Biological Research*—*Thessaloniki* in 2019—your generosity is much appreciated and we hope your association with the journal continues in the future.

*Editor*-*in*-*Chief: Theodore J Abatzopoulos*

Aharon Oren

Alessandro Chiarucci

Alexandra Staikou

Ana Claudia Oliveira Carreira

Anargyros Moulas

Andigoni Malousi

Andriana C Kaliora

Camilla Vieira Esteves

Christopher M. Roundy

David G. Harrison

Dimitris Kardassis

Dimitris Kontoyiannis

Eirini Kanata

Eleftheria Galatou

Eleni Douni

Eleni Nikolakaki

Elissavet Georgiou

Emanuela Chiarella

Eudoxia Hatzivassiliou

Florian Goedecke

George Mosialos

Grigorios Georgolopoulos

Ilias Kappas

Ioannis A. Giantsis

Ioannis Vizirianakis

Juliette K. Tinker

Karl Duffy

Kim B. Seroogy

Kimon Moschandreou

Konstantina Psatha

Konstantinos Xanthopoulos

Kostas Feidantsis

Kostas Kougioumoutzis

Makoto Noda

Maria G Roubelakis

Maria Lambropoulou

Maria Starodubtseva

Maria Touraki

Michal Rahat

Minas Yiangou

Mohammad Reza Jabalameli

Nadia Shaban

Niel Van Wyk

Nikoleta Karaiskou

Panayotis Kaloyannidis

Salih Pasa

Sinos Giokas

Sonia López

Spyridon Gkelis

Spyros Tsiftsis

Sybille D. Reichardt

Virginia Díez-Obrero

Wanning Wang

Xian-Si Zeng

Μ. Doulberis

